# Whole-genome DNA methylation profile of female *Gobiocypris rarus* brains at three age stages

**DOI:** 10.1038/s41597-026-07270-8

**Published:** 2026-04-17

**Authors:** Yongfeng He, Xinhua Zou, Menghan Wu, Jianwei Wang

**Affiliations:** 1 Institute of Hydrobiology, Chinese Academy of Sciences, Wuhan, China; 2 State Key Laboratory of Breeding Biotechnology and Sustainable Aquaculture, Institute of Hydrobiology, Chinese Academy of Sciences, Wuhan, China; 3 University of Chinese Academy of Sciences, Beijing, China

**Keywords:** DNA methylation, Predictive markers

## Abstract

*Gobiocypris rarus* is an endemic cyprinid fish in China with a lifespan of up to nine years under artificial breeding conditions and is widely used in biological and toxicological studies. However, genome-wide DNA methylation profiles across adult aging stages in this species remain limited. In this study, brain samples from 27 female individuals at three representative adult stages (8, 44, and 74 months) were subjected to whole-genome bisulfite sequencing (WGBS). After quality filtering to remove low-quality reads, adaptor sequences, and ambiguous bases, an average of 41.36 Gb clean bases per sample was obtained. Three methylation contexts (CG, CHG, and CHH) were identified, with average methylation proportions of 79.95 ± 1.01%, 1.19 ± 0.14% and 1.13 ± 0.10%, respectively. This dataset provides a high-resolution brain methylome resource across representative adult stages of rare minnow and enables future comparative and integrative epigenetic analyses in teleost species.

## Background & Summary

Biological aging is a progressive process taking place over the whole lifespan, being accompanied by remarkable alterations at the cellular, tissue and organismal levels. The biochemistry of aging is complex, e.g., genomic instability, telomere shortening, epigenetic alterations, loss of proteostasis, deregulated nutrient sensing, mitochondrial dysfunction, cellular senescence, stem cell exhaustion, and altered intercellular communication^1^. DNA methylation is one of the most in-depth and well characterized epigenetic modifications in aging research. Many studies focus on discovering linear relationships between time and various molecular data, such as global hypomethylation, hypermethylation of CpG islands, and notably age predictors based on linear DNA methylation of mammals^2^. In addition to linear trends, more complex age-related methylation dynamics have been reported in certain contexts, highlighting the diversity of epigenetic changes during aging^3–5^.

Although fish exhibits indeterminate growth and relatively high levels of methylcytosine, several studies have demonstrated age-associated DNA methylation changes in species such as zebrafish (*Danio rerio*), *Oncorhynchus tshawytscha*, and *Oncorhynchus mykiss*, and epigenetic clocks have been developed for species including *Dicentrarchus labrax*, *D. rerio*, *Neoceratodus forsteri*, *Maccullochella mariensis*, and *M. peelii* were constructed^6–11^. However, comprehensive whole-genome methylation datasets spanning representative adult life stages remain limited in fish, particularly for brain tissues. The dynamic landscape of DNA methylation across adult aging stages in teleosts therefore remains insufficiently characterized.

Rare minnow (*Gobiocypris rarus*), being also known as juji, is an endemic cyprinid fish in China, which has been extensively used as an aquatic laboratory animal in ecotoxicology, ichthyopathology, and genetics^12^. Under controlled breeding conditions, sexual maturity is reached at approximately four months, and maximum lifespan can extend up to nine years^13^. Age-related phenotypes observed in this species, including spinal curvature, reproductive decline, reduced locomotor activity, and cognitive impairment, support its utility as a vertebrate model for aging studies. Rare minnow exhibits three representative adult stages: mature adult (4–12 months old), aging adult (24–48 months old), and aged adult (>60 months old). Furthermore, high-quality whole genome sequence of rare minnow was assembled and published in 2022, and its genome size was revealed about 1.04Gb^14^. However, the genome-wide DNA methylation profiles across adult aging stages in this species have not yet been systematically characterized.

In the present study, we generated whole-genome bisulfite sequencing (WGBS) data from brain tissues of adult female rare minnow at three representative age stages (8, 44, and 74 months), with three biological replicates per group (n = 9). This dataset establishes a high-resolution brain methylome resource spanning early adulthood to advanced age and provides a valuable foundation for future comparative and integrative analyses of age-associated epigenetic variation in teleost species.

## Methods

### Sample collection and DNA extraction

A total of 27 female *Gobiocypris rarus* individuals were collected at three different ages (8 months, 44 months, and 74 months) from the National Aquatic Biological Resource Center (NABRC). For each age group, nine individuals were sampled. For downstream methylation analysis, brains from three individuals were pooled to generate one biological replicate, resulting in three independent pooled biological replicates per age group (n = 3 per group).

Prior to pooling, biological characteristics of each individual, including total length, standard length and body weight, were measured. For each biological replicate, measurements were obtained from three individuals and presented as mean ± standard deviation (SD) (see Table 1).

**Table 1:** Biological characteristics of the sampling individuals in the study.

Sample	Number of individuals	Total length (mm)	Standard length (mm)	Body weight (g)	Group	Age (months)
A1	3	47.67 ± 1.53	39.33 ± 1.53	1.39 ± 0.31	Adult	8
A2	3	48.33 ± 2.52	40.33 ± 1.53	1.56 ± 0.23		
A3	3	47.33 ± 1.53	39.33 ± 1.53	1.36 ± 0.12		
B1	3	58.00 ± 1.73	47.67 ± 1.15	1.59 ± 0.17	Aging adult	44
B2	3	54.33 ± 4.04	44.67 ± 3.06	1.39 ± 0.31		
B3	3	54.00 ± 1.73	44.00 ± 1.73	1.55 ± 0.09		
C1	3	76.67 ± 2.08	62.67 ± 2.08	4.99 ± 2.46	Aged adult	74
C2	3	73.33 ± 4.16	61.67 ± 5.69	4.82 ± 1.58		
C3	3	68.67 ± 2.52	57.67 ± 3.51	4.20 ± 1.10		

Note: For each biological replicate, measurements were obtained from three individuals and presented as mean ± standard deviation (SD).

Genomic DNA was extracted from each pooled brain sample using the SDS method. DNA concentration was quantified using a Qubit fluorometer (Invitrogen, Carlsbad, CA, USA), and DNA integrity was assessed by agarose gel electrophoresis prior to library construction.

### Library preparation and whole-genome bisulfite sequencing

Nine DNA Libraries were prepared with MGIEasy Whole Genome Bisulfite Sequencing Library Prep Kit (BGI-Shenzhen, China). Ligated DNA was bisulfite converted using the EZ DNA Methylation-Gold kit (ZYMO). The bisulfite treated library products were amplified through PCR reaction and subjected to quality control. The single-stranded cyclized DNA products were produced, amplified with phi29 and rolling circle amplification (RCA) to make DNA nanoball (DNB). Sufficient quality DNBs were then loaded into patterned nanoarrays and sequenced through combinatorial Probe-Anchor Synthesis (cPAS) in the MGI 2000 platform (BGI-Shenzhen, China).

### Read quality analysis and alignment

Software SOAPnuke (v1.5.6) was used to perform quality filtering of raw reads. Reads were removed if they: (i) contained adaptor contamination, (ii) had > 10% ambiguous bases (N), or (iii) contained > 10% bases with quality score ≤ 20^15^. Clean reads were aligned to the rare minnow reference genome (NCBI assembly GCA_023029165.1) using BSMAP (v2.74)^16^. The following alignment parameters were applied: -u -v 8 -z 33 -p 4 -n 0 -w 20 -s 16 -f 10 -L 150. Clean rate was calculated as the proportion of clean reads after quality filtering relative to raw reads. Mapping rate represents the percentage of reads successfully aligned to the reference genome relative to clean reads, and uniquely mapped rate represents the percentage of reads uniquely aligned relative to clean reads. Bisulfite conversion rate was estimated based on the conversion efficiency of non-CpG cytosines.

### Methylation calling

Cytosine methylation levels were calculated at single-base resolution as: *R*m = Nm/(Nm+Nnm) × 100% (Nm represents the reads number of methylation reads, while Nnm represents the reads number of non-methylation reads). Only cytosine sites with coverage ≥ 5 × were retained for downstream analysis. Cytosine methylation was analyzed separately in CG, CHG, and CHH sequence contexts.

### Genomic feature annotation

Genomic annotation was performed using the GFF annotation file corresponding to assembly GCA_023029165.1, downloaded from NCBI (accessed on XX date). Gene-centric elements were defined as: 2 kb upstream of the transcription start site (Up2k), mRNA regions, coding sequence (CDS), and 2 kb downstream of the transcription termination site (Down2k). Genome-wide features included repeat regions (RepeatMasker v4.0.6 annotation) and CpG Islands predicted using cpgplot from the EMBOSS package (v6.6.0.0).

### Methylation profiling

Average methylation levels were calculated separately for CG, CHG, and CHH sequence contexts. Regional methylation levels were computed as the mean methylation level of cytosine sites within each genomic element or chromosome.

### Principal component analysis

Principal component analysis (PCA) was performed in R (version 4.5.2) using the prcomp function based on genome-wide CpG β-values. CpG sites with zero variance were removed, and the top 10,000 most variable CpG sites were retained. PCA plots were generated using the ggplot2 package.

### Differential methylation analysis

Differentially methylated regions (DMRs) were identified using metilene (v0.2-7). Regions containing at least five CpG sites with a q-value < 0.05 and a minimum coverage of five reads per CpG in each condition were considered significant.

### Statistical analysis

Statistical analyses were performed in R (version 4.5.2). Standard deviations were calculated based on biological replicates within each age group. Correlation analysis of phenotypic traits and DNA methylation were conducted in R, with *P* < 0.05 considered statistically significant.

### Ethics statement

All animal procedures were approved by the Institutional Animal Care and Use Committee of Institute of Hydrobiology, Chinese Academy of Sciences (Approval No. IHB/LL/2024033) and were conducted in accordance with national guidelines.

## Data Records

The cleaned WGBS sequencing reads are publicly available in the NCBI Sequence Read Archive (SRA) under accession number SRP598880, associated with BioProject PRJNA128812317^17^. Processed methylation data generated in this study have been deposited in Figshare^18^. The dataset includes genome-wide methylation matrices for CG, CHG, and CHH contexts, chromosome-level and genomic feature-level methylation summaries, as well as tables of differentially methylated regions (DMRs) identified across age groups.

## Technical Validation

### Data quality control

To ensure sufficient data quality for downstream methylation analysis, sequencing quality filtering and alignment metrics were evaluated using SOAPnuke and BSMAP (Table 2). On average, WGBS sequencing generated 41.36 Gb of clean bases per sample after quality filtering. The proportion of clean reads with Q20 scores exceeded 94.26% across all samples, indicating high sequencing accuracy. The bisulfite conversion rate was greater than 99.26% for all samples, supporting the reliability of single-base resolution methylation detection.

**Table 2:** Read yield, filtering and mapping statistics for nine WGBS libraries.

Sample	Clean reads (number)	Q20 (R1, %)	Q20 (R2, %)	Clean Rate (%)	Mapping Rate (%)	Uniquely Mapping Rate (%)	Bisulfite Conversion Rate (%)	Mean depth (×)
A1	315981546	96.59	97.52	92.98	84.37	72.56	99.41	29.31
A2	313257102	96.60	97.27	94.58	84.54	72.96	99.45	29.11
A3	274933414	94.28	96.11	93.27	82.89	71.36	99.33	25.28
B1	275710116	94.74	95.67	93.03	82.76	71.69	99.31	25.42
B2	266384926	95.71	95.98	92.03	83.57	72.01	99.32	25.11
B3	290243854	95.04	96.29	92.74	83.52	72.23	99.33	27.51
C1	228323074	94.71	95.62	92.94	82.97	71.57	99.26	21.31
C2	230000038	95.89	97.38	93.19	83.83	72.42	99.35	21.81
C3	286903956	94.26	96.22	92.01	82.31	70.92	99.28	26.67

Note:

All libraries were constructed with paired-end 150 bp sequencing and fragment sizes of 100–500 bp.

Clean reads were obtained after adapter trimming and quality filtering.

Q20 values represent the percentage of bases with a Phred quality score ≥ 20 for read 1 (R1) and read 2 (R2), respectively.

Clean rate was calculated as the proportion of clean reads after quality filtering relative to raw reads.

Mapping rate represents the percentage of reads successfully aligned to the reference genome relative to clean reads.

Uniquely mapped rate represents the percentage of reads uniquely aligned to the reference genome relative to clean reads.

Bisulfite conversion rate was estimated based on the conversion efficiency of non-CpG cytosines.

Mean depth (×) indicates the average sequencing depth per CpG site across the genome.

### Sample clustering and reproducibility

**Fig. 1:**
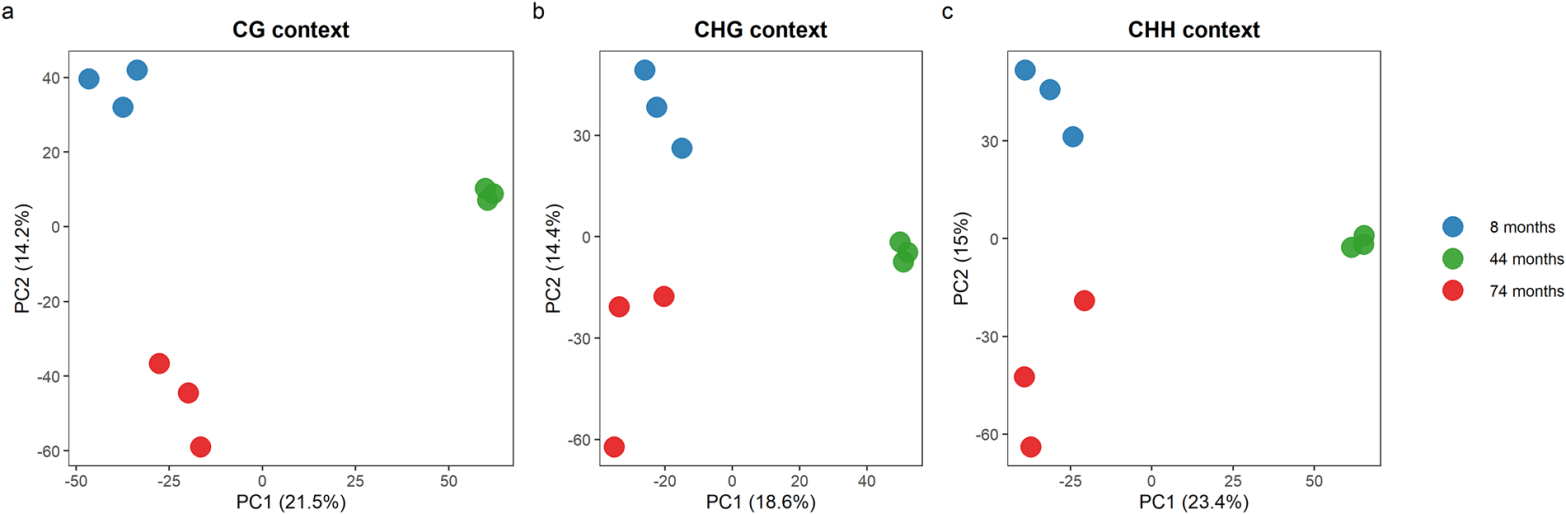
Principal component analysis of genome-wide DNA methylation across age groups. Principal component analysis (PCA) was performed on whole-genome bisulfite sequencing (WGBS) methylation data to assess global methylation variation among samples from different age groups. Prior to PCA, CpG sites with zero variance across samples were removed, and the top 10,000 most variable CpG sites were selected. PCA was conducted using the prcomp function in R with scaling enabled. (**a**) PCA based on CG methylation levels. (**b**) PCA based on CHG methylation levels. (**c**) PCA based on CHH methylation levels. Each point represents one biological sample, and colors indicate different age groups. The percentages on the axes represent the proportion of variance explained by the first two principal components.

Principal component analysis (PCA) based on genome-wide CG, CHG, and CHH methylation levels showed clear clustering of biological replicates within each age group, supporting the reproducibility of the dataset (Fig. 1). The first two principal components explained 21.5% and 14.2% of the variance for CG methylation, 18.6% and 14.4% for CHG methylation, and 23.4% and 15.2% for CHH methylation.

## Data Overview

### Global methylation landscape

Genome-wide DNA methylation levels were first assessed across all samples. The overall proportion of methylated cytosines (mC) ranged from 9.66% to 10.26%, with an average of approximately 9.99% across the nine brain samples. When stratified by sequence context, CG methylation showed the highest level, ranging from 78.29% to 80.95% among samples. In contrast, non-CG methylation was much lower, with CHG and CHH methylation levels ranging from 0.97–1.35% and 0.95–1.25%, respectively. These results indicate that DNA methylation in rare minnow is predominantly enriched in the CG context.

### Genomic feature-specific methylation patterns

**Fig. 2:**
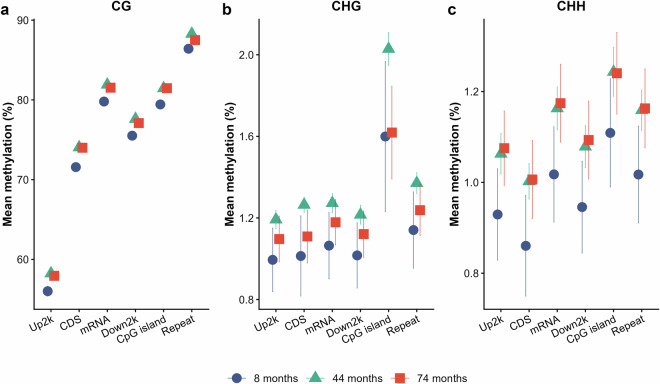
Age-related variation in DNA methylation levels across genomic features in different sequence contexts. Mean DNA methylation levels in the CG (**a**), CHG (**b**), and CHH (**c**) sequence contexts across six genomic features, including upstream 2 kb regions (Up2K), coding sequences (CDS), mRNA regions, downstream 2 kb regions (Down2K), CpG islands, and repetitive elements. Each point represents the mean methylation level for a given genomic feature in samples at 8 months, 44 months, and 74 months. Error bars indicate the standard deviation of methylation levels among biological replicates. Distinct symbols denote age groups: circles (8 months), triangles (44 months), and squares (74 months).

DNA methylation patterns varied across different genomic features (Fig. 2). In the CG context, mean methylation levels differed markedly among genomic features, ranging from approximately 56% in the upstream 2 kb region (Up2k) to nearly 89% in repetitive elements. Coding regions (CDS) and gene bodies (mRNA) showed relatively high methylation levels, whereas upstream and downstream flanking regions displayed comparatively lower methylation. CpG islands exhibited intermediate CG methylation relative to other genomic features. In contrast, CHG and CHH methylation levels were substantially lower than CG methylation, generally remaining below 2%, while exhibiting broadly similar feature-associated distribution patterns across genomic regions. Error bars represent standard deviations derived from biological replicates.

### Chromosome-level methylation distribution

**Fig. 3:**
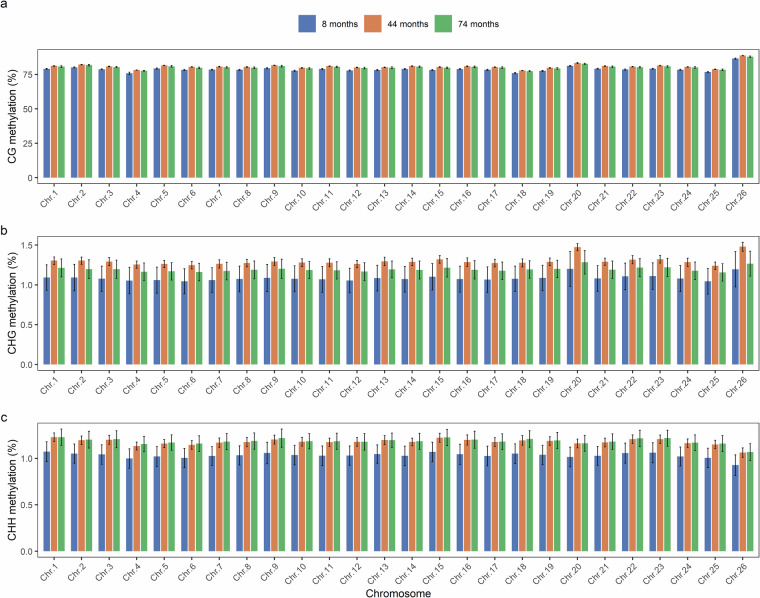
Chromosome-wide DNA methylation levels in different age groups. Chromosome-level DNA methylation profiles derived from whole-genome bisulfite sequencing (WGBS) data for three age groups (8, 44, and 74 months). (**a**) Average CG methylation levels across chromosomes. (**b**) Average CHG methylation levels across chromosomes. (**c**) Average CHH methylation levels across chromosomes. Bars represent the mean methylation level (%) for each chromosome, and colors denote different age groups. Error bars indicate the standard deviation among biological replicates.

Genome-wide methylation levels were further examined at the chromosome level (Fig. 3). CG methylation levels were consistently high across all chromosomes, generally ranging from approximately 76% to 89% among the three age groups. In contrast, CHG and CHH methylation levels were substantially lower, with values typically ranging from about 1.0–1.5% for CHG and 0.9–1.3% for CHH. Although moderate variation in methylation levels was observed among individual chromosomes, the overall context-dependent pattern was highly consistent across chromosomes and age groups. Error bars represent standard deviations calculated from biological replicates, indicating relatively small within-group variability.

### DMR summary

**Fig. 4:**
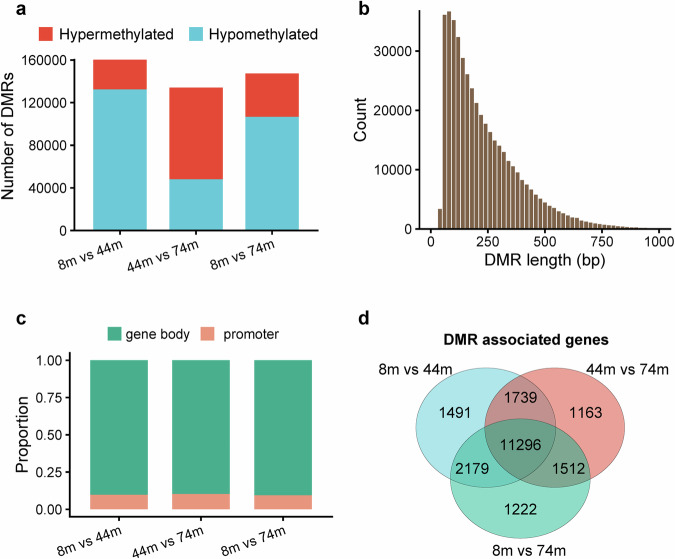
Genome-wide characteristics of differentially methylated regions (DMRs) among three age groups (8, 44, and 74 months). (**a**) Numbers of hypermethylated and hypomethylated DMRs identified in pairwise comparisons. (**b**) Length distribution of DMRs across the genome. The x-axis represents DMR length (bp), and the y-axis indicates the number of DMRs in each length interval. (**c**) Proportional distribution of DMR-associated genes located in promoter and gene body regions for each age-group comparison. Promoter regions were defined as 2 kb upstream of transcription start sites. (**d**) Venn diagram showing the overlap of DMR-associated genes among pairwise comparisons (8 m vs 44 m, 44 m vs 74 m, and 8 m vs 74 m).

Pairwise comparisons among the three age groups (8, 44, and 74 months) identified substantial numbers of differentially methylated regions (DMRs) (Fig. 4). A total of 160,263 DMRs were detected between the 8- and 44-month groups, including 27,928 hypermethylated and 132,335 hypomethylated regions. Between the 44- and 74-month groups, 134,078 DMRs were identified, whereas 147,311 DMRs were detected between the 8- and 74-month groups. The identified DMRs ranged from 49 bp to 2,203 bp in length, with a median length of 189 bp, and contained an average of six CpG sites. Most DMRs were located within gene bodies, whereas only a small proportion overlapped promoter regions. In total, 20,602 genes were associated with DMRs. Because non-CG methylation levels were extremely low in this species, DMR identification was restricted to the CG context.

### Correlation analysis of phenotypic traits and DNA methylation

To characterize the relationships between phenotypic traits, age, and DNA methylation, correlation analyses were performed. Total length, standard length, and body weight were all significantly positively correlated with age (Pearson correlation, *P* < 0.05). No significant correlations were observed between phenotypic traits and DNA methylation levels, including both global and context-specific methylation metrics. Global methylation levels (Global mC) and CG methylation (mCG) were significantly positive correlation with age (*P* < 0.05), whereas non-CG methylation (mCHG and mCHH) showed no significant associations. Correlation results are provided in Supplementary Table S1.

## Usage Notes

This dataset provides a genome-wide DNA methylation resource for rare minnow brain across three age stages. It may facilitate the development of epigenetic age predictors in fish, support comparative analyses of methylation dynamics across tissues or environmental conditions, and enable integrative studies combining methylome and transcriptome data to investigate potential relationships between DNA methylation and gene expression during brain aging.

## Supplementary information

Supplementary Table S1

## Data Availability

The cleaned WGBS sequencing reads are available in the NCBI Sequence Read Archive (SRA) under accession number SRP598880, associated with BioProject PRJNA128812317. Processed methylation data have been deposited in Figshare and are publicly available at 10.6084/m9.figshare.31769062. All files can be accessed and downloaded without restriction.

## Code availability

Data processing relied on SOAPnuke and BSMAP. Downstream statistical analyses and visualization were performed in R (version 4.5.2). All analysis scripts are available on GitHub (https://github.com/He-Yongfeng/rare_minnow_brain_methylome_analysis) and archived in Zenodo (10.5281/zenodo.19108131).
